# Synaptic microarchitecture: the role of spatial interplay between excitatory and inhibitory inputs in shaping dendritic plasticity and neuronal output

**DOI:** 10.3389/fncel.2024.1513602

**Published:** 2024-12-20

**Authors:** Dario Cupolillo, Vincenzo Regio, Andrea Barberis

**Affiliations:** Istituto Italiano di Tecnologia, Synaptic Plasticity of Inhibitory Networks, Genova, Italy

**Keywords:** excitatory synapses, inhibitory synapses, dendritic integration, dendritic spikes, heterosynaptic plasticity, dendritic modeling, pyramidal neurons, interneurons

## Introduction

Pyramidal neurons (PNs) receive and integrate 1,000's of synaptic inputs impinging onto their dendritic arbor to shape the neuronal output. The richness and the complexity of such input-output transformation primarily relies on the ability of neurons to generate different forms of dendritic local spikes, regenerative events originating in the dendrites profoundly influencing the probability and the temporal structure of somatic spiking. Extensive work during the last two decades has identified the impact of clustering and cooperative plasticity among glutamatergic synapse in promoting dendritic spikes. However, the role of inhibitory synapses in such processes remains elusive. In this opinion paper, following a general introduction on the impact of the synaptic input spatial distribution in neuronal activity, we highlight the coordinated plasticity of excitatory and inhibitory dendritic synapses as an emerging key factor in the organization of the dendritic input architecture. In particular we will emphasize that the relative positioning of diverse excitatory and inhibitory dendritic synapses at the microscale level is a major determinant for shaping dendritic dynamics and neuronal circuit function in the brain.

## Multiscale spatial arrangement of dendritic excitatory or inhibitory synaptic inputs

**Figure 1:**
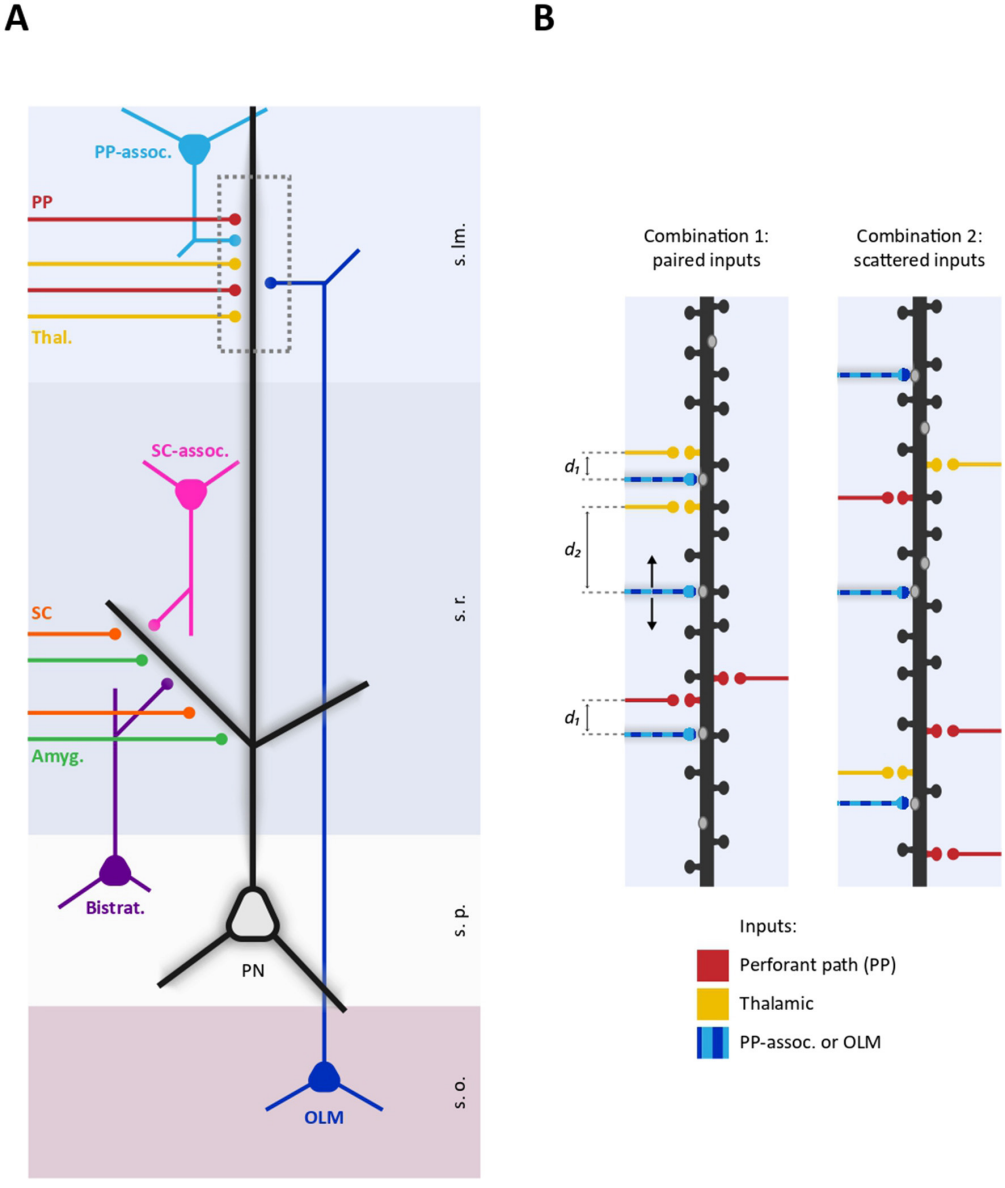
Schematic representation of proximo-distal dendritic compartmentalization of diverse GABAergic and glutamatergic inputs on a CA1 pyramidal neuron. **(A)** Representative selection of excitatory and inhibitory inputs received by CA1 dendrites. Specific subsets of excitatory inputs are aligned with distinct GABAergic fibers. Proximal dendrites in the stratum radiatum are targeted by SC (orange), amygdala projections (green), as well as local SC-associated interneurons (pink) and bistratified interneurons (purple). In contrast, distal dendrites in the stratum lacunosum moleculare receive inputs from the thalamus (yellow), the EC through the PP (red), O-LM interneurons (dark blue), and PP-associated interneurons (light blue). Dashed box delineates a distal dendritic portion represented in **(B)**. **(B)** Two different possible spatial arrangements of excitatory and inhibitory inputs on a distal dendritic segment. **(Left)** GABAergic inputs from either O-LM or PP-associated interneurons (striped light-dark blue) are positioned within an “interplay range” with thalamic or PP inputs (d1) or located beyond this range (d2). This points to the existence of excitatory-inhibitory spatial combinations, wherein certain inhibitory inputs consistently spatially paired with specific subsets of glutamatergic inputs. **(Right)** Excitatory and inhibitory inputs are randomly distributed along a dendritic segment. In this spatial arrangement, there are no consistent rules determining the pairing of specific GABAergic and glutamatergic inputs at the microscale level (s.o., stratum oriens; s.p., stratum pyramidale; s.r., stratum radiatum; s.lm., stratum lacunosum moleculare; PP, perforant path; SC, Scaffer Collaterals; Thal, Thalamic; Amyg, Amygdala).

In different brain areas, distinct synaptic inputs converging onto PNs show a macro-scale distribution across large dendritic compartments. For instance, in the hippocampal formation, excitatory fibers from entorhinal cortex (EC) project to the distal portions of apical dendrites of CA1 PNs through the perforant path (PP), while Schaffer collaterals (SCs) from the CA3 area mainly contact the proximal dendrites (Megías et al., 2001; Figure 1). Similarly, in the neocortex, intra-cortical layer 2/3 (L2/3) PNs axons (feedforward information) contact the proximal dendrites of layer 5 (L5) PNs, with cortico-cortical inputs from high-order cortical areas (feedback information) targeting their distal dendrites (Larkum, 2012). This illustrates a large-scale connectivity scheme wherein fibers from either distant or local brain regions preferentially contact distal or proximal dendrites, respectively (Felleman and Van Essen, 1991). Such broad-scale input organization reflects important functional properties where the activation of proximal dendrites typically produces single action potentials while co-activation of distal and proximal synaptic inputs can generate calcium plateau potentials—specific forms of dendritic spikes initiated in the distal dendritic region—leading the neuron to burst firing (Jarsky et al., 2005; Takahashi and Magee, 2009; Larkum et al., 1999). This supra-linear integration provides the biophysical basis for a fundamental associative process to combine and compare different types of information at the single cell level (Bittner et al., 2015; Larkum, 2012). Along the same line, the differential effect of distal feedforward inputs triggering single spikes and the combined activation of feedforward and distal feedback inputs inducing burst firing, provides the opportunity for the independent transmission of these two distinct signals through the same neuronal pathway (multiplexing; Naud and Sprekeler, 2017).

Intriguingly, GABAergic inputs are also non-randomly distributed along the axo-dendritic axis of PNs. Diverse subclasses of GABAergic interneurons (INs) target specific sub-regions of PNs including axon initial segment, soma, proximal dendrites and distal dendrites, with a specific temporal activation critically contributing to e.g., brain oscillations (Klausberger and Somogyi, 2007; Tzilivaki et al., 2023). In both hippocampus and neocortex, the proximo-distal dendritic compartmentalization of diverse GABAergic inputs creates a spatial pattern where distinct GABAergic fibers broadly align with specific subsets of excitatory inputs. For example, in the hippocampus, oriens-lacunosum-moleculare (O-LM), neurogliaform, and perforant path (PP)-associated INs target the distal dendrites of CA1 pyramidal neurons aligning with PP inputs from the EC. Comparably, bistratified, SC-associated and Ivy interneurons match glutamatergic inputs from CA3 onto proximal dendrites (Klausberger, 2009; Lovett-Barron et al., 2012; Figure 1).

The existence of structured patterns of synaptic inputs localization persists at smaller scales. At glutamatergic side, computational and experimental works showed that dendritic synaptic inputs clustering favors dendritic spikes initiation (Mel, 1993; Poirazi and Mel, 2001; Poirazi et al., 2003a,b; Larkum et al., 2009). In L5 PNs, for instance the activation of glutamatergic inputs within a ~ 40 μm range undergo supra-linear summation due to N-methyl-D-aspartate (NMDA) receptor-dependent regenerative mechanism, whereas inputs more than 80 μm apart integrate linearly, indicating the key role of the spatial determinants in dendritic input summation (Polsky et al., 2004). The functional clustering of glutamatergic inputs has been observed directly in dendrites of both CA3 and L2/3 PNs, where spontaneous activity is more likely to co-activate neighboring glutamatergic spines rather than distant spines, thus forming glutamatergic synaptic “assemblets” within ~ 10 μm (Takahashi et al., 2012). The clustered organization of glutamatergic inputs underpins an important role at the functional level. In the visual cortex, the clustering of similarly tuned inputs aids edge detection and contour integration (Iacaruso et al., 2017), while in the motor cortex, task-related inputs cluster within 10 μm subdomains to support decision-making (Kerlin et al., 2019). Besides the relevance of the tight spatial proximity between active glutamatergic synapses (*synaptic clustering*), the initiation of dendritic spikes strongly depends on the dendritic morphology. In thin and short dendritic branches, the high input resistance determines low attenuation of the depolarization produced by individual synapses thus promoting the signal summation within the branch (Kastellakis and Poirazi, 2019). For instance, the timely activation of ~ 20 glutamatergic inputs on a radial oblique dendritic branch of 100 μm in CA1 PNs initiate a local sodium spike regardless of their spatial relationship along the branch, thus determining *in-branch clustering* (Losonczy and Magee, 2006). Anatomical studies of SCs synapses localization onto CA1 PNs dendrites have revealed a highly non-uniform connectivity structure. In particular, the number of short inter-spine distances as well as the number of glutamatergic inputs per branch was greater than chance level, thus supporting both synaptic clustering and in-branch clustering modes, respectively (Druckmann et al., 2014). Similar findings were observed for thalamocortical inputs onto L5 PN (Rah et al., 2013). Collectively, this evidence indicates that, at different scales, the spatial arrangement of glutamatergic synapses in dendrites of PNs crucially shapes the transfer function between synaptic activation and dendritic depolarization/spiking (Ujfalussy and Makara, 2020; Kastellakis and Poirazi, 2019). It is interesting to note that, synaptic inputs in dendrites of interneurons are less spatially structured with respect to PNs (Kwon et al., 2018), and, in contrast to PNs, synaptic inputs in small caliber dendrites of fast spiking basket cells tend to summate sub-linearly (Tzilivaki et al., 2019).

As with excitatory inputs, several lines of evidence show that synaptic inhibition in PNs dendrites depends on local spatial determinants at the microscale level, such as their fine relative positioning with respect to excitatory synapses (Boivin and Nedivi, 2018). Modeling studies suggest that GABAergic synapses positioned distally (off-path) from a cluster of glutamatergic synapses more efficiently raise the threshold for initiating a dendritic spike compared to proximally-placed ones (on-path), whereas the on-path location is more effective in shunting already-triggered dendritic spikes (Gidon and Segev, 2012). Both predictions have been corroborated experimentally *ex vivo* in L5 PNs, confirming that the specific spatial arrangement of GABAergic synapses in dendritic branches is an important determinant shaping dendritic excitability (Jadi et al., 2012). In this concern, studies report that diverse GABAergic inputs from specific interneurons are highly structured at branch and sub-branch levels. In CA1 PNs, O-LM interneurons (somatostatin+, SOM+) or neurogliaform interneurons (neural nitric oxide synthase+, nNOS+) preferentially target the ending or the intermediated region of the terminal domain of distal dendrites, respectively whereas bistratified interneurons (neuropeptide Y+, NPY+) target the origin of the terminal domain of proximal apical oblique and basal dendrites (Bloss et al., 2016). In addition, the study of the excitatory and inhibitory synapses distribution in the whole dendritic arbor in L2/3 PNs revealed that, while density of both synapses significantly vary in different neuronal sub-regions, its ratio was remarkably balanced at branch level (Iascone et al., 2020). Finally, inhibitory GABAergic synapses can be located directly on glutamatergic spines thus effectively controlling spine depolarization (Boivin and Nedivi, 2018; Chiu et al., 2013).

## How does cooperative plasticity among glutamatergic synapses shape synaptic clustering?

Extensive work on glutamatergic spines reports that the expression of long-term potentiation (LTP) at an individual spine can lower the threshold for the induction of synaptic plasticity at neighboring spines by spreading signaling molecules such as small GTPases from the potentiated spine in dendritic stretches of ~10 μm: this establishes the coordinated potentiation of a subset of contiguous spines ultimately leading to the formation of a glutamatergic synaptic cluster (Harvey and Svoboda, 2007; Harvey et al., 2008; Hedrick and Yasuda, 2017). On the other hand, the stimulation of a glutamatergic spine cluster can depress nearby spines through the diffusion of the phosphatase calcineurin, a mechanism that is expected to increase the structural and functional identity of specific clusters (Oh et al., 2017). Likewise, long-term depression (LTD) at an individual spine can either depress or potentiate neighboring spines (Chater and Goda, 2021). Overall, these observations suggest that short-range interplay between spines can define the spatial pattern of dendritic glutamatergic synapses. Nevertheless, how GABAergic synapses contribute to these processes remains largely obscure. Traditionally, inhibition has been considered poorly plastic and to take part to plasticity phenomena mainly by adjusting the threshold for the induction of glutamatergic plasticity (Steele and Mauk, 1999). In this concern, modeling studies report that specific placement of GABAergic synapses with respect to either excitatory synapses or dendritic branches can spatially constraint glutamatergic plasticity hence influencing the degree of glutamatergic synapses clustering (Bar-Ilan et al., 2012). Similarly, the activation of GABAA receptors by GABA uncaging leads to the shrinkage of nearby glutamatergic spines within a range of ~15 μm, reinforcing the spatial role of inhibition in promoting the competitive selection of dendritic spines (Hayama et al., 2013).

Nevertheless, several lines of evidence indicate that GABAergic synapses express several forms of plasticity (Chiu et al., 2019). This prompts the questions of how glutamatergic and GABAergic plasticity interact at dendritic level at the microscale level and how this can shape synaptic clustering—topics that have thus far been investigated mainly through indirect approaches (Chapman et al., 2022). After the induction of spike-timing-dependent plasticity at a specific synaptic population subset in an auditory cortex PN, the plasticity of excitatory and inhibitory plasticity at distinct unstimulated synaptic population subset was found to be co-tuned to achieve a precise excitation-to-inhibition set point (Field et al., 2020). Interestingly, the plasticity-induced remodeling of excitatory and inhibitory synapses on dendrites of L2/3 PNs in the visual cortex is spatially coordinated in dendritic portions of ~10 μm suggesting short-range interplay between inhibitory and excitatory synapses (Chen et al., 2012). In addition, the stimulation of thalamic afferents to distal dendrites of cortical L2/3 PNs induces inhibitory LTP at GABAergic synapses formed by SOM+ interneurons in the same dendritic portion, thus hinting to local interaction between excitatory and inhibitory synapses (Chiu et al., 2018). Extending this framework, a modeling study identifies the presence of plastic GABAergic synapses as important organizers of dendritic glutamatergic synaptic clustering (Kirchner and Gjorgjieva, 2022).

A more recent work investigated the spatial determinants for the interaction between individual dendritic glutamatergic and GABAergic synapses in hippocampal neurons (Ravasenga et al., 2022). By inducing single-spine LTP through the pairing of glutamate uncaging with somatic action potential train, they observed that GABAergic synapses located within a spatial range of ~3–4 μm around the potentiated spine were depressed. Although several factors could limit the generalization of this finding including the poorly physiological induction of LTP and the lack of *in vivo* data, the spatial dependence of the interaction between excitation and inhibition likely plays an important role in the organization of dendritic synaptic inputs. First, by considering the local effect of inhibition (Gidon and Segev, 2012), this heterosynaptic interplay is expected to disinhibit specific potentiated glutamatergic inputs through a winner-takes-all process, with e.g., other concurrent plasticity phenomena maintaining the global dendritic homeostatic balance. Second, the activity-dependent depression of a neighboring GABAergic synapse can contribute to the formation of glutamatergic synaptic clusters thus complementing the cooperative plasticity phenomena between glutamatergic inputs mentioned above. Finally, in the light of this short-range interplay, the convergence of diverse excitatory and inhibitory inputs within the same dendritic stretch can crucially impact at the network level, allowing, for instance, specific glutamatergic inputs to differentially control inputs from different interneuron subtypes. For example, PP and thalamic inputs contact the distal apical dendrites of CA1 PNs together with inputs from O-LM and PP-associated interneurons, which primarily mediate feed-back and feed-forward inhibition, respectively. If, differently from thalamic inputs, EC inputs are consistently located within the “interplay range” with inputs from O-LM interneurons, EC activity could weaken neighboring O-LM inputs (Figure 1). This could bias the balance of inhibition from feedback to feed-forward, thereby altering how these dendrites process and integrate incoming signals. Thus, in analogy with the aforementioned large-scale matching between excitation and inhibition in proximal and distal dendritic compartments, it is important to define the co-alignment between excitatory and inhibitory inputs at the microscale level. The spatial pattern of diverse excitatory and inhibitory inputs along the dendrites may serve as a “fingerprint” for PN subtypes, where the consistent pairing of particular excitatory inputs with inhibitory inputs from specific interneurons could act as structural “synaptic motifs.” In a broader framework, the impact of excitatory-inhibitory short-range synaptic interplay can be assessed by including specific synaptic topology and plasticity rules in available biophysical computational models predicting the spiking output of PNs receiving realistic excitatory and inhibitory temporal activity patterns at cellular level. This will allow to understand how short-range plasticity contribute to modulate specific network oscillations by tuning at dendritic level the contribution of diverse interneuron subtypes, or how it could enable associative learning by differentially gating information from distinct brain areas. Importantly, this could also clarify how aberrant short-range plasticity could lead to the disruption of coordination between different interneurons subtypes activity ultimately causing pathology. In the long run, the refined information about the dendritic synaptic spatial arrangement and short-range interaction could be integrated in computational models that include dendritic computation in large-networks functions and will also contribute designing more neuromorphic and efficient deep neuronal networks (DNNs; Pagkalos et al., 2024, 2023).
